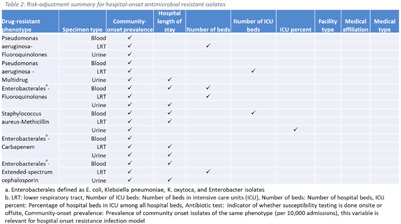# Developing national benchmarks for antimicrobial resistance–NHSN, 2019

**DOI:** 10.1017/ash.2022.182

**Published:** 2022-05-16

**Authors:** Hsiu Wu, Erin O’Leary, Minn Soe, Jonathan Edwards

## Abstract

**Background:** The emergence and spread of drug-resistant pathogens continues to significantly impact patient safety and healthcare systems. Although antimicrobial susceptibility test (AST) results of clinical specimens are used by individual facilities for antimicrobial resistance surveillance, accurate tracking and benchmark comparison of a facility’s antimicrobial resistance using national data requires risk-adjusted methods to be more meaningful. The CDC NHSN Antimicrobial Resistance (AR) Option collects patient-level, deduplicated, isolate information, including AST results, for >20 organisms from cerebrospinal fluid, lower respiratory tract (LRT), blood, and urinary specimens. To provide risk-adjusted national benchmarks, we developed prediction models for incidence of hospital-onset isolates with antimicrobial resistance. **Methods:** We analyzed AST results of isolates reported through the NHSN AR Option for January through December 2019. Isolates from facilities that had >10% missing AST results for the organism-drug combinations or from hospitals that used outdated breakpoints were excluded. We assessed associations between facility-level factors and incidence rates of hospital-onset (specimen collected 3 days or more after hospital admission) isolates of specific drug-resistant phenotypes from blood, LRT, and urinary specimens. Factors included number of beds, length of stay, and prevalence of community onset isolates of the same phenotype. Drug-resistant phenotypes assessed included methicillin-resistant *Staphylococcus aureus* (MRSA), multidrug-resistant (MDR) *Pseudomonas aeruginosa*, carbapenem-resistant Enterobacterales (CRE), fluoroquinolone-resistant *Pseudomonas aeruginosa*, fluoroquinolone-resistant Enterobacterales, and extended-spectrum cephalosporin-resistant Enterobacterales. Isolates of different phenotypes and from different specimen sources were modeled separately. Negative binomial regression was used to evaluate the factors associated with antimicrobial resistance incidence. Variable entry into the models is based on significance level P Among the models, 1 for each drug-resistant phenotype-specimen type combination, the number of isolates with AST results ranged from 718 (*Pseudomonas aeruginosa*–fluoroquinolones, blood) to 16,412 (Enterobacterales–fluoroquinolones, urine). The pooled incidence rate was highest for fluoroquinolone-resistant Enterobacterales in urinary specimens (0.2179 isolates per 1,000 patient days) among all phenotype-specimen combinations evaluated (Table 1). The incidence of drug-resistant isolates was consistently associated with community-onset prevalence across models evaluated. Other associated factors varied across phenotype-specimen combinations (Table 2). **Conclusions:** We developed statistical models to predict facility-level incidence rates of hospital-onset antimicrobial resistant isolates based on community-onset drug-resistant prevalence and facility characteristics. These models will enable facilities to compare antimicrobial resistance rates to the national benchmarks and therefore to inform their antimicrobial stewardship and infection prevention efforts.

**Funding:** None

**Disclosures:** None

**Table:**
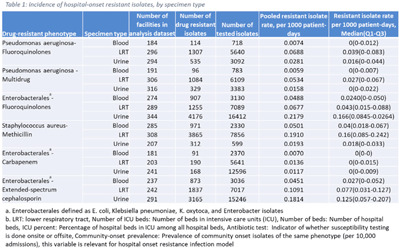

**Table:**